# Querying phenotype-genotype relationships on patient datasets using semantic web technology: the example of cerebrotendinous xanthomatosis

**DOI:** 10.1186/1472-6947-12-78

**Published:** 2012-07-31

**Authors:** María Taboada, Diego Martínez, Belén Pilo, Adriano Jiménez-Escrig, Peter N Robinson, María J Sobrido

**Affiliations:** 1 Department of Electronics and Computer Science, University of Santiago de Compostela, Edificio Monte da Condesa, Campus Vida, 15782, Spain; 2 Department of Applied Physics, University of Santiago de Compostela, Santiago de Compostela, Spain; 3 Section of Neurology, Hospital del Sureste, Arganda del Rey, Madrid, Spain; 4 Department of Neurology, Hospital Ramon y Cajal, University of Alcalá de Henares, Alcalá de Henares, Spain; 5 Institut für Medizinische Genetik und Humangenetik, Charité - Universitätsmedizin, Charité, Berlin, Germany; 6 Fundación Pública Galega de Medicina Xenómica, Santiago de Compostela. Center for Biomedical Research on Rare Diseases (CIBERER), Institute of Health Carlos III, Santiago de Compostela, Spain

## Abstract

**Background:**

Semantic Web technology can considerably catalyze translational genetics and genomics research in medicine, where the interchange of information between basic research and clinical levels becomes crucial. This exchange involves mapping abstract phenotype descriptions from research resources, such as knowledge databases and catalogs, to unstructured datasets produced through experimental methods and clinical practice. This is especially true for the construction of mutation databases. This paper presents a way of harmonizing abstract phenotype descriptions with patient data from clinical practice, and querying this dataset about relationships between phenotypes and genetic variants, at different levels of abstraction.

**Methods:**

Due to the current availability of ontological and terminological resources that have already reached some consensus in biomedicine, a reuse-based ontology engineering approach was followed. The proposed approach uses the Ontology Web Language (OWL) to represent the phenotype ontology and the patient model, the Semantic Web Rule Language (SWRL) to bridge the gap between phenotype descriptions and clinical data, and the Semantic Query Web Rule Language (SQWRL) to query relevant phenotype-genotype bidirectional relationships. The work tests the use of semantic web technology in the biomedical research domain named cerebrotendinous xanthomatosis (CTX), using a real dataset and ontologies.

**Results:**

A framework to query relevant phenotype-genotype bidirectional relationships is provided. Phenotype descriptions and patient data were harmonized by defining 28 Horn-like rules in terms of the OWL concepts. In total, 24 patterns of SWQRL queries were designed following the initial list of competency questions. As the approach is based on OWL, the semantic of the framework adapts the standard logical model of an open world assumption.

**Conclusions:**

This work demonstrates how semantic web technologies can be used to support flexible representation and computational inference mechanisms required to query patient datasets at different levels of abstraction. The open world assumption is especially good for describing only partially known phenotype-genotype relationships, in a way that is easily extensible. In future, this type of approach could offer researchers a valuable resource to infer new data from patient data for statistical analysis in translational research. In conclusion, phenotype description formalization and mapping to clinical data are two key elements for interchanging knowledge between basic and clinical research.

## Background

*Cerebrotendinous xanthomatosis* (*CTX*) is a rare lipid-storage disease that leads to a complex combination of neurologic dysfunctions including cerebellar, pyramidal and extrapyramidal signs, neuropathy, dementia and psychiatric disturbances, as well as extra-neurological manifestations (chronic diarrhea, cataracts, tendon xanthomas, premature arteriosclerosis) [1]. CTX is caused by mutations in the gene *CYP27A1*, which encodes the mitochondrial enzyme sterol 27-hydroxylase, deficiency of which causes an excess of intermediate metabolites such as cholestanol to accumulate in virtually every tissue. Like many neurodegenerative disorders, CTX is characterized by an insidious onset, progressive course and variable combination of clinical manifestations in each patient, which, together with the rarity of the disease, hampers correct and early diagnosis. Therapeutic delay is especially catastrophic in CTX, since there is a specific treatment (chenodeoxycolic acid), which is effective in reducing the plasma levels of cholestanol but has not been demonstrated to improve established neurological deficits. Mutation analysis of the *CYP27A1* gene is a key step in the diagnosis of CTX and is routinely performed. The availability of comprehensive genotype to phenotype data sets will be crucial in order to promote early recognition and optimize the diagnostic process.

In any disease but most especially in rare diseases the possibility of accessing detailed patient datasets from research and clinical studies, including genetic variants and phenotypic manifestations, would significantly improve diagnosis and treatment. Electronic patient records are able to gather diverse types and growing amounts of phenotypic data, while the use of genome-scale DNA sequencing techniques allows the collection of an increasing number of genetic variants per individual. Thus, integrating complex phenotype descriptions with genetic testing records has become one of the main challenges of biomedicine [2]. As the number of openly accessible datasets continues to rise, the integration of research repositories and patient clinical data will be more viable. However, bioinformatics tools are needed to help explore complex genotype-phenotype relationships. Geneticists would request software tools able to retrieve and analyze the data produced in diverse clinical settings and associated to a new given genetic variant; that is, answering questions like *what are the phenotype traits that have been identified in patients with this genetic variation?* Clinicians, on the other hand, would see their work greatly facilitated by being able to answer queries like *what genes or genetic variants are associated with this particular combination of observable features?*

The development of locus-specific mutation databases (LSDBs) and tools to build them such as the Leiden Open Variation Database (LOVD) [3], and the Universal Mutation Database (UMD) [4] started to pave the way to solve the problem of collecting genetic datasets produced by diverse experimental methods in different laboratories. However, the phenotype description in most LSDBs is very scarce. The Human Variome Project (HVP) [5] is an international initiative aiming ultimately at the worldwide collection and harmonization of all human genetic variations and associated phenotypic data. The GEN2PHEN project also represents an international attempt to undertake the logistical and technical challenges to join disparate genotype-phenotype resources in a shared mode [6]. In order to achieve that goal, communication standards are needed to allow interoperability between clinical and genetic datasets. Standards to represent genetic findings are already available, such as those produced by the HUGO committee (http://www.genenames.org/aboutHGNC.html), gene relationships provided by Gene Ontology [7] or the nomenclature for description of sequence variants proposed by the Human Genome Variation Society (HGVS, http://www.hgvs.org/mutnomen). However, such a level of consensus on the best descriptors for phenotypic information is far more complex and has not been reached in clinical medicine.

Although the term *phenotype* covers an extensive range of information varying from molecular to organism level observable characteristics [8], in this work phenotype is meant only as any observable human trait, such as an anatomical abnormality (e.g., j*uvenile cataracts*) or a clinical feature *(*e.g.*, tendon xanthomas*). Currently, the most useful catalog of human Mendelian disorders is OMIM, the Online Mendelian Inheritance in Man [9], a text-based knowledge source of human phenotypes and related genes. OMIM describes phenotypes using narrative sentences (e.g. *normal to slightly elevated plasma cholesterol*). Although these textual descriptions are highly expressive, capturing phenotype information using free-text fields in databases hampers computational processing and inference [10]. The use of a standard terminology provides a more appropriate method of expressing unambiguous, computable, and interoperable phenotype descriptions. Standard terminologies organize the concepts of a particular domain into a taxonomy (e.g., e*pilepsy* is a *seizure disorder*, which is an *abnormality of the central nervous system*), assigning them identifiers which do not change with new versions. They also address the issues of different synonyms for the same concept (e.g., *convulsions* vs. *epileptic seizures*).

Patient data from clinical and research settings are usually stored in different formats, from simple spreadsheets to relational databases, being extremely difficult to integrate genotype-phenotype data across multiple formats. The semantic web technology provides an adequate instrument of recording phenotypes in a standardized fashion and with a high degree of expressivity. Using this technology to represent data will ensure the compatibility of them with the future knowledge and data resources. Additionally, one of the main challenges of articulating queries on phenotype-genotype relationships is discrepancy in the level of abstraction between phenotype descriptions and patient clinical data. The semantic web technology provides a layer of abstraction that makes it simple to use. Moreover, this technology is based on *open world* assumption: everything we do not know is undefined. Hence, unknown relationships will be interpreted as not computable instead of false. This approach naturally deals with incomplete information, which is very usual in biomedicine, and it is able to refine knowledge when new information comes along.

One option to deal with the phenotype complexity can be to define a minimum set of phenotype template fields [11]. In contrast, an ontology-based technology would provide a more open and flexible representation mechanism [10,12-14], thus facilitating the continuing incorporation and interpretation of new phenotype characteristics. An ontology is a data model that represents a set of entities in some domain and the relationships among those entities. One of the benefits of using ontologies is the potential to apply reasoners (logical inference tools), which can infer new data to subsequently facilitate query answering and statistical analysis. In the present work, we used patient data from a specific rare genetic disease (CTX) to formally represent phenotype descriptions using the ontological paradigm [15]. We then engineered the patient data in an ontology-based patient model and finally executed queries on genotype-phenotype relationships with a Semantic Web approach.

## Methods

### Overview of the query process

**Figure 1:**
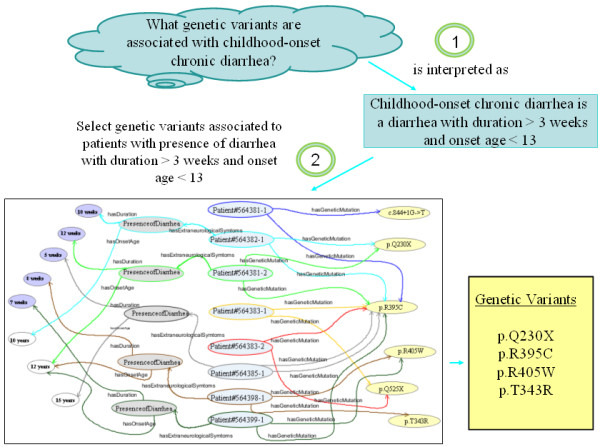
**Steps required to answering questions on phenotype-genotype relationships.** The figure includes an example showing the genetic variants that have been identified in patients with childhood-onset chronic diarrhea (i.e., p.Q230X, p.R395C, p.R405W and p.T343R).

Since the main goal of our work was to develop an approach to query genotype-phenotype relationships on patient datasets, we set out a list of competency questions [16], i.e., questions that our approach should be able to answer. Then, we analyzed the query process in order to determine the steps required to optimize the answers. The list of competency questions included queries such as the one showed in Figure 1. These queries are expressed with phenotype descriptions like those used in genetic catalogs (e.g., *childhood-onset chronic diarrhea*). Patient clinical data, however, usually contain this information in a lower level of abstraction, often distributed in several fields (e.g., *diarrhea onset age* and *diarrhea duration*). Thus, a tool provided with a query like the one in Figure 1 should carry out, at least, two steps: first, successfully interpreting the meaning of *childhood-onset chronic diarrhea* and second, adequately mapping the query to the dataset.

Additionally, queries such as ‘*What genetic variants have been identified in patients without childhood-onset chronic diarrhea?*’ will return those genetic variants for patients with asserted absence of *childhood-onset chronic diarrhea*, that is, the genetic variant *p.R395C* (patient *564385*–*1*) in Figure 1. But, it will not include those patients for which there is unknown or missing information (open-world assumption); that is, it will say “Do not know” for the genetic variants *c.844 + 1 G- > T* y *p.Q525X*, unless a blank is interpreted as ‘not (*childhood-onset chronic diarrhea)’.*

**Figure 2:**
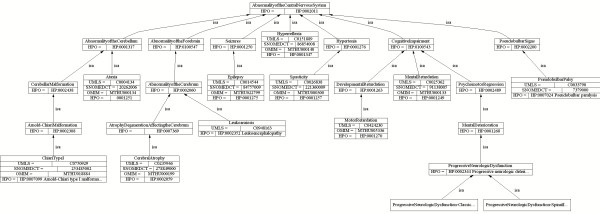
Example of hierarchical (‘is-a’) relationships between Abnormalities of the Central Nervous System and more specific disorders, such as Epilepsy or Ataxia.

Finally, if phenotype descriptions are organized in an *is_a* hierarchy, queries for broader phenotypes should return genetic variants associated with these phenotypes as well as with narrower phenotypes. For example, the query *Select genetic variants associated with Abnormality of the Cerebellum* should also return genetic variants associated with *Ataxia and Arnold-Chiari type I malformation* (Figure 2).

### Data and knowledge sources

Patient Datasets: The complete and detailed patient dataset published on a PhD dissertation written in Spanish [17] was used in our approach. Currently, these data are also available in English in a more summarized form [18]. These patient data originated from a thorough collaborative clinical and genetic study on CTX carried out at the Hospital Ramón y Cajal (Madrid) and the Fundación Pública Galega de Medicina Xenómica (Santiago de Compostela) in Spain.

In brief, 25 patients from 19 families were thoroughly studied through personal examination of the patients and their medical records by the authors (BP, AJE, MJS). Detailed clinical history data, neurological signs, neurophysiologic, biochemical and neuroimaging data were collected in extensive table and text format.

Text-based resources: Two text-based resources from highly reliable web sites were searched in our approach, the OMIM clinical synopsis (http://www.ncbi.nlm.nih.gov/omim/213700?dopt=Synopsis; [19]) and the Summary, Diagnosis and Clinical Description sections of GeneReviews (http://www.ncbi.nlm.nih.gov/bookshelf/br.fcgi?book=gene&part=ctx), both of them containing known phenotype manifestations of CTX.

Terminology systems: The Unified Medical Language System (*UMLS¸*http://umlsks.nlm.nih.gov*)* Metathesaurus was chosen to codify the relevant CTX terminology extracted from the text-based resources.

Domain ontologies: Two web sites actively used in biomedical communities, the NCBO Bioportal (http://bioportal.bioontology.org; [20]) and the OBO Foundry (http://www.obofoundry.org; [21]), were accessed to search ontologies and terminologies in the specific domain of CTX.

### Ontology development

**Figure 3:**
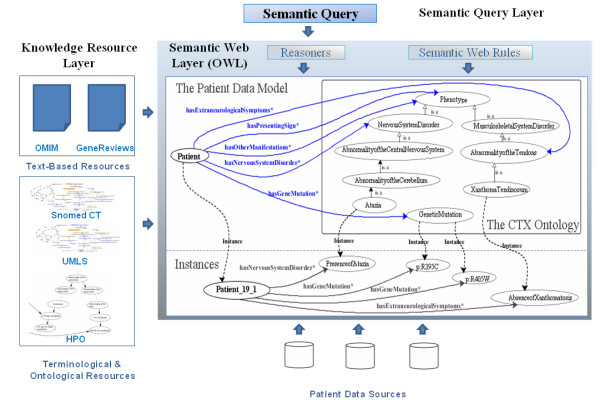
Layers of the approach proposed in this work.

Our approach distinguishes three levels (Figure 3): the Knowledge Resource Layer, in which several resources in different formats can be found; the Semantic Web Layer, which provides the domain terminologies, ontologies and data, as well as, inference and deductive capabilities to increase the usability and reusability of data; and the Semantic Query Layer, which supplies tools and languages for semantic query processing.

CTX phenotype-genotype relationships are represented in the Semantic Web Layer via a patient data model expressed in terms of a CTX phenotype ontology. We developed this ontology following the reuse-based methodology NeOn [16], thanks to the current availability of ontological and terminological resources that have reached some consensus in biomedicine. Although NeOn is focused on collaboratively building ontology networks, it is currently the only one covering complex scenarios with reuse of ontological and non-ontological resources. Initially, a set of use cases describing phenotype-genotype bidirectional relationships and query support were specified in order to determine the content of our ontology. Thus, the ontology should represent CTX phenotypes, genetic variants, and bidirectional relationships between them. Next, we developed the CTX ontology carrying out several activities proposed by the NeOn methodology: extracting the CTX terminology, assessing the publicly available resources, reusing and reengineering the selected resources. The ontology was developed using the Web Ontology Language (OWL) [22] and Protégé-OWL 3.4 [23].

### Extracting the CTX terminology

One of the critical steps when developing ontologies is to identify the particular knowledge to be represented in the ontology. Usually, this activity is carried out at the beginning of the ontology project through a set of interviews with domain experts. Since this activity is labor-intensive, we decided to semi-automatically extract the glossary of CTX terms from the different resources employed in this work. Then, we manually checked the extracted terminology, and we eliminated erroneously extracted concepts, very general concepts (e.g., *Findings*) and some terms that did not represent concepts relevant to CTX.

### Assessing terminological and ontological resources

We searched the term *Cerebrotendinous Xanthomatosis* in the NCBO Bioportal and in the OBO Foundry resources. Next, we assessed the candidate ontologies from these resources by calculating their coverage with respect to the glossary of CTX terms.

### Reusing terminological and ontological resources

We built the CTX ontology by reusing concepts and relationships from the selected resources in the last step.

### Construction of patient data model in OWL

We developed an OWL model describing the patient dataset and the relationships between phenotypic and genotypic data, where the former are expressed in terms of the CTX phenotype ontology.

### Querying phenotype-genotype bidirectional relationships

In order to have the ability to execute queries at different levels of abstraction, firstly a set of rules was defined to successfully interpret the meaning of phenotype descriptions (step 1 in Figure 1). This set of rules was implemented by means of the Semantic Web Rule Language (SWRL) [24], a language to express Horn-like rules in terms of OWL concepts. Next, in order to formulate queries traversing bidirectional phenotype-genotype relationships on patient data (step 2 in Figure 1), we used SQWRL [25], a language that expands SWRL in order to provide querying of OWL ontologies.

## Results

### The CTX ontology

#### Extracting the CTX terminology

Table 1 summarizes the quantitative results obtained from extracting the glossary of CTX terms using different terminological services provided by the UMLSKS Knowledge Server (ExactMatch, NormalizedString and Metamap; [26]). After manually revising the extracted terminology, the glossary of relevant terms for CTX comprised a total of 93 UMLS concepts.

**Table 1:** Percentage of match of CTX terminology extracted from different sources

	**Strings/Sentences #**	**Precision**	**Recall**	**F-Measure**
Patient Data (ExactMatch)	65	93 %	67 %	78 %
Patient Data (ExactMatch + MetaMap)	65	94 %	94 %	94 %
OMIM (NormalizeString)	23	100 %	100 %	100 %
GeneReviews (MetaMap)	83	88 %	79 %	83 %

The F measure represents 2 (P R)/(P + R), the geometric mean of the precision (P) and recall (R), which is a standard measure for the goodness of information retrieval from documents.

### Assessing terminological and ontological resources

Upon searching the term *Cerebrotendinous Xanthomatosis* in the NCBO Bioportal, 15 ontologies were displayed, some of them being collateral to the domain, such as DermLex, a terminology of the dermatologic domain, or *RadLex*, a lexicon in the radiology domain. We selected Snomed CT, as it is a large clinical terminology system containing formal definitions for clinical concepts using hierarchical and non-hierarchical relationships. We then examined the OBO Foundry resources and here two ontologies related to the phenotype domain were selected as candidates: the Human Phenotype Ontology (HPO) [27], covering human phenotypic abnormalities, and the Phenotypic Quality (PATO) [10], involving phenotypic qualities necessary to reach a complete description of phenotypes. Table 2 shows the coverage of relevant CTX terminology in the candidate ontologies, itemized by Disorders and Abnormalities, Anatomical Structures, Diagnostic Studies and Qualifier Values.

**Table 2:** Coverage of relevant CTX terminology in Snomed CT, HPO and PATO

	**Disorders & Abnormalities (Concept #)**	**Anatomical Structures (Concept #)**	**Diagnostic Studies (Concept #)**	**Qualifier Values (Concept #)**
Snomed CT	56	6	11	11
	(93.3 %)	(100 %)	(92 %)	(73.3 %)
HPO	50	----	----	-----
	(83.3 %)			
PATO	----	----	----	5
				(33.3 %)
Total	60	6	12	15
	(100 %)	(100 %)	(100 %)	(100 %)

### Reusing terminological and ontological resources

Although the coverage of relevant CTX terminology in Snomed CT was slightly higher than in HPO (Table 2), we decided to reuse HPO and extend it with the required Snomed CT concepts and relationships. The justification is the following: the organization of HPO follows the structure of OMIM, which is very close to experts in CTX, HPO is less complex than Snomed CT and it can be easily translated to OWL.

All concepts were integrated within the target application as phenotype management ontology, consisting of four main hierarchies: *Phenotype, Anatomical Structure, Diagnostic Studies and Qualifier Values*. Table 3 shows the total number of concepts in the OWL ontology (279), detailing the number of terms pulled from HPO and SNOMED CT, as well as the number of new concepts (those not included in either HPO or Snomed CT). Reusing classes and hierarchies greatly reduces the effort needed by medically qualified personnel, which only participated in curation tasks (deciding the most suitable hierarchy in case of multiple options, positioning a new concept and revising the complete ontology).

**Table 3:** Descriptive statistics on the origin of CTX ontology concepts

	**HPO Concept #**	**SNOMED CT Concept #**	**New Concept #**
Phenotype	153	17	6
Anatomical Structure	0	42	0
Diagnostic Study	0	30	0
Qualifier Value	0	31	0
Total	153	120	6
	(55 %)	(43 %)	(2 %)

In addition, it was necessary to distinguish among the *presence* and the *absence* of a phenotypic trait in a given patient. Therefore, we subdivided each phenotype into two OWL instances covering the two possibilities. For example, for the phenotype *Epilepsy*, there are two possible instances: *presence of epilepsy* and *absence of epilepsy*. Hence, there are three types of patients: those ones with asserted presence of epilepsy, those ones with asserted absence of epilepsy, and finally those ones for which there is no information about the presence or absence of epilepsy.

### The patient data model

We created object properties to represent patient manifestations: gene mutations, extra-neurological symptoms, neurological symptoms, results from diagnostic studies and other manifestations. Then, the patient model was populated manually. In total, 1 class, 17 properties and 25 individuals represent the Patient Model in our ontology.

### Querying phenotype-genotype bidirectional relationships

Before formulating queries traversing phenotype-genotype relationships, we defined a set of 28 rules to deduce abstract phenotype descriptions from the patient data. Supplementary data provides all rules defined in our system. The execution of the rules generates the corresponding links (e.g., *has extra-neurological manifestations’*) between the corresponding patient instances and the abstract phenotype instances. Next, we formulate some of the queries defined in the initial list of competency questions, showing how the approach can help us to analyze the CTX dataset. (Supplementary data provides the complete list of queries, showing the corresponding SQWRL implementation and the answers in every case).

Example 1: In the CTX study, the presence of *childhood-onset chronic diarrhea* was confirmed in 44 % of patients. The reply to the query *‘What genetic variants are associated with childhood-onset chronic diarrhea?’* substantiates that only 7 from 14 genetic variants (50 %) are associated with this manifestation. Moreover, the answer to *‘What are the genetic variants that have always been associated with childhood-onset chronic diarrhea?’* verifies that only one genetic variant (*p.R395C*) is associated with both the presence and absence of the manifestation, whereas the remaining 6 genetic variants are always associated with the presence of this manifestation. Our approach also provides queries like ‘*What are the genetic variants that have never been associated with childhood-onset chronic diarrhea?.* In the CTX study, only the generic variant *p.Q525X* was never associated.

Example 2: In the CTX study, the presence of *epilepsy* was corroborated in 32 % of patients, and the presence of *dementia* in 52 % of patients. The answer to *‘What genetic variants are associated with epilepsy and dementia at the same time?’* confirms that only 6 from 14 genetic variants (42 %) are associated (see Table 4).

**Table 4:** Four examples of queries to the patient data

***Pattern of CQ***	***Examples of CQ***	***Example of SQWRL***	***Example of answers***
*What are the genetic variants that have been associated with a combination of traits?*	What are the *genetic variants* that have been associated with *epilepsy* and *dementia*?	Patient(?p) ^ hasNervousSystemDisorder(?p, ?x) ^ Epilepsy(?x) ^ hasPresence(?x, ?y) ^ Yes(?y) ^ hasNervousSystemDisorder(?p, ?d) ^ Dementia(?d) ^ hasPresence(?d, ?y) ^ hasGeneMutation(?p, ?g) ^ GeneticMutation(?g) ^mutation(?g, ?m) → sqwrl:columnNames("GeneMutation") ^ sqwrl:selectDistinct(?m)	c.844 + 1 G- > T
			p.N403K
			p.R395C
			p.R405W
			p.T339M
			p.T343R
*What are the abnormalities that have been associated with a specific genetic variant?*	What are the Abnormalities of the Central Nervous System that have been associated with *p.R395C*?	Patient(?p1) ^ hasNervousSystemDisorder(?p1, ?z) ^ hasPresence(?z, ?y) ^ Yes(?y) ^ AbnormalityoftheCerebellum(?z) ^ hasGeneMutation(?p1, ?g) ˚ sqwrl:makeSet(?s1, ?z) ^ Patient(?p2) ^ hasOtherManifestations(?p2, ?x) ^ hasPresence(?x, ?y) ^ AbnormalityoftheCerebellum(?x) ^ hasGeneMutation(?p2, ?g) ^ sqwrl:makeSet(?s2, ?x) ^ GeneMutation(?g) ^ mutation(?g, ?m) ^ swrlb:equal(?m, "p.R395C") ˚ sqwrl:append(?s3, ?s1, ?s2) ^ sqwrl:element(?e, ?s3) → sqwrl:select(?e)	PresenceofAtaxia
			PresenceofChiariTypeI
*How often has a specific genetic variant been associated with a specific trait?*	How often has *p.R395C* been associated with *Ataxia*?	Patient(?p1) ^ hasNervousSystemDisorder(?p1, ?x) ^ Ataxia(?x) ^ hasPresence(?x, ?y) ^ Yes(?y) ^ hasGeneMutation(?p1, ?g) ^ GeneMutation(?g) ^ mutation(?g, ?m) ^ swrlb:equal(?m, "p.R395C") ˚ sqwrl:makeSet(?s1, ?p1) ˚ sqwrl:size(?size1, ?s1) ^ Patient(?p2) ^ hasGeneMutation(?p2, ?g) ^ sqwrl:makeSet(?s2, ?p2) ^ sqwrl:size(?size2, ?s2) ^ swrlb:multiply(?mu, ?size1, 100.0) ^ swrlb:divide(?d, ?mu, ?size2) → sqwrl:select(?d)	57 %
*What is the average number of years from the onset of a symptom to the onset of another symptom (or diagnosis/death) in patients with a given genetic variant?*	What is the average number of years from the onset of diarrhea to the first neurological symptom in patients with the genetic variant p.R395C?	Patient(?p1) ^ hasGeneMutation(?p1, ?g) ^ GeneticMutation(?g) ^ mutation(?g, "p.R395C") ^ hasDiarrheaAge(?p1, ?d) ^ AgeatFirstSymptom(?d) ^ age(?d, ?da) ^ hasNeurologicalSymptomsOnsetAge(?p, ?a) ^ AgeatFirstSymptom(?a) ^ age(?a, ?ca) ^ swrlb:subtract(?di, ?ca, ?da) → sqwrl:columnNames("Average age from diarrhea to neurological symptoms onset") ^ sqwrl:avg(?di)	7 years

The first query is about genetic variants associated with a specific combination of observable features; the second query is about phenotype traits associated to a specific genetic variant; and the third and fourth ones are examples of querying information about frequency and elapsed time associated with the presence of a specific genetic variant and trait.

Example 3: The study compiled the set of main neurological manifestations and their frequency. The request *‘What are the central nervous system manifestations that have been identified in patients with p.R395C?’* supplies us with the specific manifestations (Ataxia and Chiari Type I) for a particular genetic variant (see Table 4).

Example 4: The CTX study linked the onset age of neurological symptoms with the presence of *xanthomas*. In patients with *xanthoma*s, the average onset age of neurological symptoms was 23 years in contrast with 16 years for patients without *xanthomas*. Inquiring about *What is the average onset age of nervous system symptoms in patients with xanthomas and p.R405W?* returns 31 years in contrast with 23,4 years (almost 8 years before) when the same query is made for the genetic variant *p.R395C.* Similarly, the query *What is the average onset age of nervous system symptoms in patients without xanthomas and with p.R395C?* confirms 12,7 years (more than 3 years before the general average of patients). The system provides a specific query to ask this type of questions directly: *How long after the onset of xanthomas the first neurological symptom appears in patients with the genetic variant p.R395C compared to all patients?*

Table 4 shows two other examples of queries to the patient data. Supplementary data provide the complete set of 24 patterns of SWQRL queries, which were designed following the competency questions. The results obtained for all queries in the ontology were tested with manual analysis during implementation. If a query did not return the expected result (from manual analysis), we assumed that the query was formulated wrongly and so, it was changed until to get the manual result.

## Discussion

Our approach shows how the ontological paradigm and the semantic web languages OWL, SWRL and SQWRL can be used in combination to develop tools to explore phenotype-genotype bidirectional associations in the particular clinical domain of CTX, a rare, autosomal-recessive neurometabolic disease. Two quite similar approaches were previously demonstrated in familial hypercholesterolemia [28] and autism cases [29]. In the first one, an ontology was used to guide the expert with the choice of meaningful subsets of a large mass of genomic and post-genomic data. In the second one, a specific domain ontology was developed and complemented with an information model and a rule-based inference function, to infer high-level phenotypic abstractions. An interesting alternative to define some of these rules is the use of OWL 2, a new version of OWL, which considerably improves the OWL data types. Using OWL2, concepts such as *Childhood-onset chronic diarrhea* will be directly expressible with OWL2 using data type restrictions to define ranges for the concepts. Another option to define some SQWRL queries is to create defined OWL classes (through asserting necessary and sufficient conditions) and to use the reasoner to automatically compute the inferred types.

Although the present work is limited to a very specific domain and with data collected from only a limited number of patients through a Spanish research study, it shows the potential use in other rare diseases and larger datasets. One of the main problems for the correct diagnosis and handling of rare genetic disorders is the difficulty in recognizing their variable clinical expression, as well as the early symptoms of the disease. We propose here an example of the type of tools that can aid clinical practice by querying about potential genetic causes of a specific symptom combination. The power of this type of tools will greatly depend on both the amount and quality of data that nurture the system. Thus, two sets of advancements are extremely necessary in order to improve diagnosis and treatment of genetic diseases, especially for rare diseases: 1) Promoting international initiatives to gather large sets of accessible patient data and 2) Developing phenotype ontologies and bioinformatics tools, in order to query phenotype-genotype relationships. Achieving this necessary level of detailed genotype and phenotype description while handling patient datasets appropriately to protect individual confidentiality, is a question that is being addressed by the Human Variome Project consortium ethics committee [30].

The approach proposed here is a pilot model that must be taken with due caution, especially with small datasets. Variable penetrance and expressivity are common in neurogenetic disorders and phenotype trait description of a given patient will evolve with time. Thus, phenotype-genotype correlations are certainly not absolute. That is, we cannot assert that a patient with a specific mutation will necessarily have the same phenotype as another patient stored in the database with the same mutation. Still, if used with awareness of its limitations, this type of genotype-phenotype exploration will be of clinical utility. Furthermore, if the database is big enough it might be interesting to derive risk figures, although this is not at all easy to do directly in OWL/SQWRL.

Although the procedure used to populate the CTX ontology in this work was manual, this is not the most suitable method to incorporate new patients to the patient model. Managing this step is important for further use of the ontology or work inspired in this approach. Incorporating an ontology-based workflow to annotate patient data resources automatically, as proposed by [31], will provide the way of combining the new resources with the existing CTX ontology. Additionally, temporal dimensions to phenotypic data are important for this clinical domain and competency questions. Modeling of timelines and temporal occurrence of events is one of the most challenging problems in ontology development. The patient data used in this study only included temporal events referred to the patient age when a given manifestation had taken place (e.g., age onset of cataract) or temporal intervals referred to the duration of some manifestations. Even with this limited information, some relevant queries on temporal aspects of phenotype-genotype relationships could be designed. For example, ‘On average, how long was it from the onset of chronic diarrhea to the first neurological symptom in patients with CTX?’, ‘and in patients with CTX and the mutation p.395 C?’ Another aspect to be improved in the future is to explore the possibility of designing more complex SWRL rules dealing with probabilistic classification in order to deduce abstract phenotypes.

Our approach could also have limitations if the number of patient data increases extremely. Scalability issues or the slowness of OWL stores and reasoners are some of the disadvantages of using such a young technology [13]. Even so, there are some ways of speeding up searches in OWL. One interesting way is the use of the OWL 2 EL (i.e. existential logic) subsets [32] to enable more efficient reasoning and complex queries to support scientific analyses. Although OWL EL is focused on large-scale ontologies, exploring the OWL EL expressivity power, which is more limited than OWL, for representing and processing our approach, could be an interesting area for future research. Another current limitation to the use of this technology is the scarcity of publicly available data sources. Although we cannot anticipate the format of data sources in the future, even in the case they were available in traditional databases and not in OWL, this technology could be used by implementing a bridge between the relational patient databases and the phenotype ontology.

Currently two communities are making a big effort to supply a full and comprehensive representation of the clinical domain: The International Health Terminology Standards Development Organization (IHTSDO), with the Snomed CT common vocabulary, and the Open Biomedical Ontologies (OBO) Foundry initiative [21], providing an ontology repository covering different domains of biomedicine. A recent study analyzing both communities concluded that it is premature to know whether one or the other will supply the solution to the breadth of coverage [33]. Thus, instead of choosing between these two strategies to represent the particular CTX domain we selected the ontology HPO from the OBO Foundry initiative, and extended it with Snomed CT from IHTSDO and a limited number of newly created terms. It should be emphasized that the goal of this work was not to build an exhaustive and definitive ontology for CTX, but to develop semantic web tools to query phenotype-genotype relationships. An extra benefit of this approach is that the ontology can then be reused for other applications, such as differential diagnosis [34].

## Conclusions

Our study shows that the Semantic Web paradigm provides the technology required to represent phenotype-genotype relationships in diseases with complex and variable manifestations such as CTX. We developed a tool to query this type of relationships on patient data in both directions and at different levels of abstraction. While ontologies have been generally used in medicine to describe unambiguous and standard terminologies agreed by consensus, our approach makes use of the ontological paradigm and semantic web technologies to provide a structured framework to query about individuals presenting a combination of phenotype traits or carrying specific genetic variants. The proposed approach implies querying the patient data by designing a patient data model in OWL and accessing them via the phenotype ontology. A semantic web rule language allowed us to infer phenotypic abstractions from patient data and provided the required bridge between phenotypic abstractions and clinical data. Through the use of this language, queries about phenotype-genotype relationships can now be formulated on the abstraction level that is common in genetic databases. We believe that our strategy is a promising approach for translational medical research, which will help improve diagnosis and thus early and effective treatment of genetic disorders. This is especially true for rare diseases, where the number of affected individuals is small and therefore easy data access and query is essential to the health care community. Coordinated international initiatives such as the HVP are crucial to promote the development of the necessary tools as well as to provide openly accessible patient data.

### Availability and requirements

The CTX ontology in OWL and all information relevant to the paper is provided in following link: http://www.usc.es/keam/CTX/TheCTXOntology.html, which contains

· The complete list of Competency Questions (QC).

· Several diagrams showing the main hierarchies of the CTX phenotype management ontology.

· The complete list of SWRL rules implemented in our approach.

· The complete list of patterns of queries implemented in our approach. These groups of queries were designed following the list of CQ.

· The patient model and an example of a fictitious patient.

## Competing interests

The author(s) declare that they have no competing interests.

## Authors’ contribution

MT – created, designed and programmed the reported technical advance, drafted and revised the submitted manuscript, and gave final approval of the published manuscript. DM –designed, and programmed the reported technical advance, participated in drafting and revising the submitted manuscript, and gave final approval of the published manuscript. BP – participated in the creation and design of the reported technical advance, participated in drafting and revising the submitted manuscript, and gave final approval of the published manuscript. AJE – participated in the creation and design of the reported technical advance, participated in drafting and revising the submitted manuscript, and gave final approval of the published manuscript. PNR – participated in the creation and design of the reported technical advance, participated in drafting and revising the submitted manuscript, and gave final approval of the published manuscript. MJS – created, designed and evaluated the reported technical advance, drafted and revised the submitted manuscript, and gave final approval of the published manuscript. All authors read and approved the final manuscript.

## Pre-publication history

The pre-publication history for this paper can be accessed here:

http://www.biomedcentral.com/1472-6947/12/78/prepub
